# Age-Associated Decline in Dendritic Cell Function and the Impact of Mediterranean Diet Intervention in Elderly Subjects

**DOI:** 10.3389/fnut.2017.00065

**Published:** 2017-12-19

**Authors:** Sarah J. Clements, Monica Maijo, Kamal Ivory, Claudio Nicoletti, Simon R. Carding

**Affiliations:** ^1^Gut Health and Food Safety Research Programme, The Quadram Institute, Norwich Research Park, Norwich, United Kingdom; ^2^Faculty of Medicine and Health Sciences, Norwich Medical School, University of East Anglia, Norwich, United Kingdom; ^3^Department of Experimental and Clinical Medicine, University of Florence, Florence, Italy

**Keywords:** dendritic cells, Mediterranean diet, aging, cytokines, resistin

## Abstract

**Introduction:**

Aging is accompanied by increased susceptibility to infection and age-associated chronic diseases. It is also associated with reduced vaccine responses, which is often attributed to immunosenescence and the functional decline of the immune system. Immunosenescence is characterized by a chronic, low-grade, inflammatory state termed inflammaging. Habitants of Mediterranean (MED) regions maintain good health into old age; often attributed to MED diets.

**Hypothesis:**

Adoption of a MED-diet by elderly subjects, in Norfolk (UK), may improve immune responses of these individuals and in particular, dendritic cell (DC) function.

**Experimental approach:**

A total of 120 elderly subjects (65–79 years old) recruited onto the Nu-AGE study, a multicenter European dietary study specifically addressing the needs of the elderly, across five countries, and were randomized to the control or MED-diet groups, for one year. Blood samples were taken pre- and post-intervention for DC analysis and were compared with each other, and to samples obtained from 45 young (18–40 years old) subjects. MED-diet compliance was assessed using high performance liquid chromatography-with tandem mass spectrometry analysis of urine samples. Immune cell and DC subset numbers and concentrations of secreted proteins were determined by flow cytometric analysis.

**Results:**

As expected, reduced myeloid DC numbers were observed in blood samples from elderly subjects compared with young. The elevated secretion of the adipokine, resistin, after *ex vivo* stimulation of peripheral blood mononuclear cells from elderly subjects, was significantly reduced after MED-diet intervention.

**Conclusion:**

This study provides further evidence of numerical and functional effects of aging on DCs. The MED-diet showed potential to impact on the aging immune cells investigated and could provide an economical approach to address problems associated with our aging population.

## Introduction

With age there is a progressive decline in the functionality of the immune system, termed immunosenescence (1), which is defined by differences in biomarkers of the immune system between young and elderly subjects, and is associated with a detrimental outcome (2). This typically refers to a reduction in response to vaccinations and greater susceptibility to infection and age-associated disease, such as cardiovascular disease (CVD), rheumatoid arthritis, type II diabetes (T2D), and cancer (2, 3). Many age-associated diseases are accompanied by dysregulated immune function and excessive inflammation; termed inflammaging (4). “Inflammaging” is characterized by chronic production of pro-inflammatory cytokines such as IL-6, tumor necrosis factor alpha (TNF-α), and IL-1β by immune cells (1) and is often associated with immunosenescence (3). The burden of immunosenescence is becoming more apparent with a projected 2.9 million increase in the number of people with two or more long term conditions by 2018, and the high incidence of CVD and T2D in the elderly, which are associated with inflammation (5). Further investigation is therefore needed to identify potential new interventions to alleviate immunosenescence.

Dendritic cells (DCs) are one of the major antigen presenting cell populations of the immune system and are considered the only cells that are capable of activating naïve T cells. DCs can be divided into two phenotypically and functionally distinct subsets; plasmacytoid DCs (pDCs) and myeloid DCs (mDCs). pDCs that express low levels of MHC II and costimulatory molecules, and express TLRs 7 and 9 and secrete large quantities of type I IFN (6). mDCs, or conventional DCs that sense tissue injury and phagocytose and capture antigen for presentation to T cells, and migrate to lymphatic tissue to prime naïve T cell responses (6). However, recently, further subdivisions of human blood DCs have been suggested, with a total of six populations of DC identified following single-cell RNA sequencing (7). With age, a number of studies have found reductions in the number of pDCs, while the numbers of mDCs remained comparable between young and elderly individuals (8–10). The reasons for this inconsistency are not known but may be related to different definitions of age (9–11) and the use of different methods for enumerating DCs (8–10, 12). Since DCs are crucial for the generation of adaptive immune responses, it seems plausible that there may be an effect on DCs with increasing host age which would influence downstream T and B cell responses.

Whilst diet is a significant factor in maintaining a healthy immune system, conflicting evidence exists for the impact different food groups [carbohydrates, protein, fatty acids (FAs) and phytochemicals] have on the aging immune system, with both beneficial and detrimental effects observed. FAs can both increase and decrease pro-inflammatory cytokine secretion by DCs (13–15), of which the type of FA is of principal importance. In addition, short-chain fatty acids and polyphenols have been demonstrated to reduce secretion of IL-6 and IL-12 by lipopolysaccharide (LPS)-stimulated monocyte-derived DCS (MoDCs) and murine derived DCs, respectively (16–18). While there is some evidence that dietary intake can influence cytokine secretion by peripheral blood mononuclear cells (PBMCs) (19–21), this was not reproduced in other studies (22–24).

It is noteworthy that many diet-immune health studies to date, have investigated dietary components in isolation, or as encapsulated supplements. This ignores the real-life response after consumption of whole foods which needs to be considered along with any interactions occurring between different components of the diet. The relatively few whole diet studies that have assessed immune cells, including expression of adhesion markers or cytokine secretion, have been short in duration (typically 3 months) and have predominantly been conducted in Mediterranean (MED) countries such as Spain or Italy (25–27). A limited number of studies have investigated the Okinawan diet and New Nordic Diet but currently there is no evidence of immune modulation (28–31). Based on the current evidence of the positive health benefits of the MED diet in reducing cardiovascular risk factors, and expression of inflammatory mediators (25–27, 32, 33), predominantly in an elderly population, this diet has the potential to improve immune health. We have therefore investigated the impact of aging on DC populations and their functionality, in addition to the impact of dietary intervention with a MED diet for 12 months on DC parameters in a healthy, elderly population residing in Norfolk, UK. For these analyses, healthy young subjects (18–40 years) were recruited onto the Im-AGE study to compare the impact of aging on immune function. While, the Nu-AGE study, which was a multicenter European dietary study, across five countries, recruited elderly subjects at the University of East Anglia (UEA) to investigate whether changing the diet to resemble a MED diet, could influence immune functions in these individuals.

## Materials and Methods

### Participant Recruitment

Forty-five young participants (18–40 years) were recruited to the Im-AGE study via the Norfolk and Norwich University Hospital phlebotomy department (REC reference 15/SW/0038) and were apparently healthy and free from current or recent (3 months) chronic disease. Exclusion criteria included recent changes to medications, type I diabetes (T1D), use of steroids or immunomodulatory medication, or antibiotic use either currently or within the previous 2 months. A total of 120 elderly participants (65–79 years) were recruited to the Nu-AGE project via the Clinical Research and Trials Unit (CRTU) at the UEA (REC reference 12/EE/0109) and were apparently healthy and free from current or recent (3 months) chronic disease. Sixty subjects were enrolled into each arm of the dietary intervention. Exclusion criteria included recent changes to medications, T1D, using steroids or, taking antibiotics currently or within the previous 2 months. Both the Im-AGE and Nu-AGE studies were conducted in full compliance with the principles of the declaration of Helsinki (2013 version) and following good clinical practice. There were no conflicts of interest in relation to these studies.

### Subject Demographics

Nu-AGE study pre-baseline data were collected as a self-reported 7-day food diary (7DD), a general questionnaire to provide socioeconomic information, followed by anthropometric measurements at baseline at the UEA CRTU. Data collection was repeated 1-year post-intervention. To improve accuracy of the diet diaries, photographs of common portion sizes and servings such as mugs, glasses, and spoons were provided. Additionally, a member of the study team attended a home visit to more accurately assess portion sizes and typically consumed brands of foods or foods which may not be clearly identified in the diet diary. For the Im-AGE study a lifestyle questionnaire was completed by all participants.

### Control and MED Diets

The study participants were randomly allocated into two groups, control and intervention; the control group were provided with a standard healthy living advice leaflet from the British Dietetic Association and asked to maintain their habitual dietary intake. The participants within the intervention group were provided with dietary advice sheets and individual dietary advice by members of the study team at the CRTU at UEA to achieve the quantitative requirements for the Nu-AGE dietary intervention (Table S1 in Supplementary Material). This advice was based on the information provided within the 7-day food records collected at baseline. Study participants randomized to the dietary intervention arm of the study were given extra virgin olive oil, wholegrain pasta and low fat margarine rich in MUFA and PUFA, freely throughout the study. The study team distributed these products to intervention participants at baseline and 4 and 8 months, when the participants attended the CRTU, either for their baseline measurements or for their interim interview questionnaires to assess blood pressure, cognitive function, and physical function; data not shown.

### Dual X-Ray Bone Densitometer (DXA) Scans

Bone mineral density (BMD) and bone composition were determined using whole body DXA scans (DXA Discovery Wi dual-energy X-ray absorbtiometer; Hologic Inc.), at pre- and post-intervention study visits (Nu-AGE study only), by a trained member of the study team and according to a standard protocol. BMD was measured at the lumbar spine and proximal femur.

### Blood Sample Collection

For both studies 23 ml blood was taken by venipuncture; 3 ml with sodium heparin and 20 ml with EDTA (BD; Bunzl Healthcare) to obtain peripheral blood and PBMCs. PBMCs were isolated using Ficoll-Hypaque density gradient centrifugation (Sigma-Aldrich, density 1.077 g/ml). For functional assays, PBMCs were stored frozen in heat-inactivated fetal bovine serum (FBS) (Biosera) containing 10% dimethyl sulfoxide (Sigma-Aldrich) at −80°C before use. Further blood samples (total volume 100 ml) were collected from all Nu-AGE volunteers at pre- and post-intervention visits to obtain plasma. The plasma was used for analysis of lipid profiles (total cholesterol, LDL cholesterol, HDL cholesterol, and triglycerides). Blood samples were processed or analyzed within 4 h of collection.

### Blood DC Enumeration

Dendritic cells were enumerated in whole blood using the Blood DC Enumeration Kit (Miltenyi-Biotec) according to the manufacturers’ instructions using the monoclonal antibodies (mAbs) CD1c-PE (Clone: AD5-8E7), CD303-FITC (Clone: AC144), CD14-PE-Cy5, CD19-PE-Cy5, and CD141-APC (Clone: AD5-14H12). As controls, isotype matched mouse IgG2a-PE, IgG1-FITC, IgG1-APC, Ig-G2a-PE-Cy5, and IgG1-PE-Cy5 antibodies were used. Flow-count™ fluorospheres (Beckman Coulter) were used to obtain absolute leukocyte counts. Data were acquired on the Beckman Coulter Cytomics FC500 MPL and the Sony EC800 with a minimum of 1,000 events acquired within the flow count fluorosphere gate, and a minimum of 100,000 events acquired within the cell gate to exclude debris. Single-stained compensation controls, prepared using Ultracomp ebeads (Affymetrix eBioscience), and non-stained control samples were run on both cytometers to allow for manual compensation to be applied to all acquired sample data. Acquired data were analyzed using FlowJo™ software (TreeStar, San Carlos, CA, USA), Version 10. Data are presented from the Sony EC800 for both the Nu-AGE and Im-AGE studies, all samples were also run on the Beckman Coulter FC500 MPL to provide a back-up machine for technical failures of the Sony EC800. For each individual sample the absolute number of pDCs and mDCs per microliter of blood was calculated by subtracting the number of cells in the DC gate for the isotype control, from the count in the DC gate for the test sample. This number, for each subset, was then divided by the count of Flow-count™ fluorospheres and multiplied by the assayed concentration of the Flow-count™ fluorospheres; calculated by the manufacturer and provided with each lot of beads.

### Functional Analysis of Blood DCs

Frozen PBMCs were thawed and cultured in tissue culture media [90% Roswell Park Memorial Institute 1,640 (Sigma-Aldrich), 10% heat-inactivated FBS (Biosera), supplemented with l-glutamine (2 mM), penicillin (100 U), and streptomycin (0.1 mg/ml) (all Lonza)] for 19 h at 37°C in 5% CO_2_ in 96 well flat bottom tissue culture plates (Sarstedt) to allow recovery. Viability of all thawed PBMC samples was determined using trypan blue viability dye exclusion (0.4%; Sigma-Aldrich) and all samples were resuspended at a final concentration of 0.5 × 10^6^ cells/200 µl; to ensure that the same number of live cells were used in each culture. Before the addition of 1 µg/ml LPS (Invivogen) and 2.5 µg/ml R848 (Invivogen), in the presence or absence of 2 µM monensin (Sigma-Aldrich) for 3 h. Non-stimulated controls were incubated with fresh tissue culture media, with or without 2 µM monensin. Cell culture supernatants were stored at −80°C before cytokine analysis. To detect cytokine synthesis by DCs, cultured cells were first incubated with Fc Receptor block (Miltenyi-Biotec) for 25 min, at 4°C in the dark and then stained with anti-CD1c-PE (Clone: AD5-8E7), anti-CD303-PE (Clone: AC144), anti-CD304-PE (Clone: AD5-17F6) (all from Miltenyi-Biotec), anti-CD3-FITC (Clone: UCHT1), anti-CD16-FITC (Clone: 3G8) (Becton Dickinson), anti-CD14-FITC (Clone: TÜK4), anti-CD19-FITC (Clone: LT19), and anti-HLA-DR-Alexa Fluor 700 (Clone: L243) (all from BioLegend) antibodies. The corresponding isotype controls IgG1-FITC (BioLegend), IgG2a-PE, IgG1-PE (Miltenyi-Biotec), and IgG2-AF700 (BioLegend) were also used. Cells were then fixed and permeabilized using Leucoperm cell fixation and permeabilization reagents (AbD Serotec), then stained for intracellular cytokines with the mAbs IL-1β-PE-Cy7 (Clone: JK1B-1), IL-6-Pacific Blue (Clone: MQ2-13A5), and CXCL8-Alexa-Fluor-647 (Clone: E8N1), or the corresponding isotype controls PE-Cy7-Mouse IgG1 and Alexa-Fluor-647-Rat IgG1 (all from BioLegend). Cells were incubated for 30 min at 21°C, in the dark, washed with PBS/0.5% FBS then resuspended in flow cytometry buffer containing 1% formalin. Data were acquired on the BD LSR Fortessa cytometer. Spectral overlap that occurred between channels was manually compensated in FlowJo™ software Version 10, after measurement of single-stained compensation controls prepared using Ultracomp ebeads (Affymetrix eBioscience) and unstained control samples. Data were analyzed using FlowJo™ software Version 10.

### Cytokine Content of Culture Supernatants

Supernatants from PBMC cultures were analyzed for CXCL8, IL-1β, IL-6, monocyte chemoattractant protein-1 (MCP-1), TNF-α, leptin, IL-10, adiponectin, adipsin, IFN-γ, IP-10 (CXCL10), RBP4, and resistin by multiplex immunoassay (LEGENDplex Human Adipokine panel; BioLegend) according to the manufacturers’ instructions. The standard provided within each kit was serially diluted (1:4) six times after the top standard (neat standard); in addition to assay buffer (zero standard). Each well contained sample or a standards dilution series and, after addition of the premixed beads and detection antibody, were resuspended in 300 µl wash buffer before acquiring data on the flow cytometer (BD Fortessa X-20). Acquired data were analyzed using the BioLegend LEGENDplex software. Briefly, standard curves were produced for each of the 13 analytes to allow the concentrations of each analyte, within the samples measured, to be determined; based on mean fluorescence intensity values relating to known concentrations for serial dilution of the standards. The standard curves also provided validation for each experiment. Inter-plate variability was determined for experiments carried out on different days.

### Statistical Analysis

Subject demographics of young and elderly subjects (at baseline) were compared using Mann–Whitney *U* tests, after carrying out D’Agostino and Pearson normality tests, using GraphPad Prism Version 7. Comparisons made between the baseline and post-intervention time points within each dietary group (control or MED-diet intervention) used paired *t*-tests. Blood mDC and pDC phenotypes were compared between elderly and young volunteers using a Welch–Satterthwaite *t*-test on rank transformed data. For mDC, the size of the difference (34) and the 95% confidence intervals (35) were estimated for a Wilcoxon Mann–Whitney *U* test. A Mann–Whitney *U* test was used to determine the presence of differences between the control and dietary intervention cohorts. GraphPad Prism Version 7 was used to determine the differences in proportions and cell counts of DCs which were positive for IL-6, CXCL8, and IL-1β secretion between young and elderly subjects using Mann–Whitney *U* tests, after performing a D’Agostino and Pearson normality test to determine non-Gaussian distribution. Proportions and cell counts of DCs positive for IL-6, CXCL8 and IL-1β secretion at pre- to post-intervention for each intervention group were compared using Wilcoxon matched pairs signed rank tests, after performing a D’Agostino and Pearson normality test to determine non-Gaussian distribution. *Post hoc* analyses were performed by one-way analysis of variance (ANOVA) using the Kruskal–Wallis test with Dunn’s multiple comparisons *post hoc* test to identify any differences in proportions of single and double positive cells. GraphPad Prism Version 7 was used to determine interplate variability between multiplex immunoassays using repeated-measures ANOVA. Differences in concentration of each analyte between unstimulated and stimulated samples were calculated using two-tailed paired *t*-tests, while differences in concentration change from unstimulated level between young and elderly subjects was determined using two-tailed Mann–Whitney *U* tests for each analyte. Differences in concentration from unstimulated were compared per group, pre- versus post-intervention using Mann–Whitney *U* tests. Comparison of change from baseline (CFB) between control and MED-diet groups was carried out using Mann–Whitney *U* tests.

## Results

### Subject Demographics of Young and Elderly Cohorts

Mean ages of the two groups were 30.71 years for the young and 70.33 years for the elderly cohort (Table 1). Both groups contained a greater number of female participants although this was not significantly different. The mean weight and BMI of participants between groups was similar though the upper range of weights of the elderly cohort was greater than for the young group. When considering height there was a significant difference between the young and elderly groups, with the elderly having a lower mean value, which is relevant as losing height with age impacts BMI.

**Table 1:** Baseline anthropometric data for Im-AGE and Nu-AGE participants.

		Im-AGE (*n* = 45)	Nu-AGE (*n* = 122)	*p* value
Age (years)	Mean (SD)	30.71 (6.36)	70.33 (4.16)	<0.0001
	Range	20–40	65–79	

Gender	% Female	57	61	0.8589 (ns)

Weight (kg)	Mean (SD)	74.46 (14.02)	73.27 (13.85)	0.6676 (ns)
	Range	53.98–108.0	49.50–128.50	

Height (cm)	Mean (SD)	169.30 (10.03)	165.70 (9.16)	0.0311
	Range	147.30–191.00	145.60–188.20	

BMI (kg/m^2^)	Mean (SD)	26.13 (3.66)	26.62 (3.96)	0.7237 (ns)
	Range	19.86–34.15	18.50–43.20	

*ns, not statistically significant; SD, standard deviation*.

### Numbers of mDCs Were Reduced in the Elderly, while pDC Numbers Remained Similar to Young Subjects

**Figure 1:**
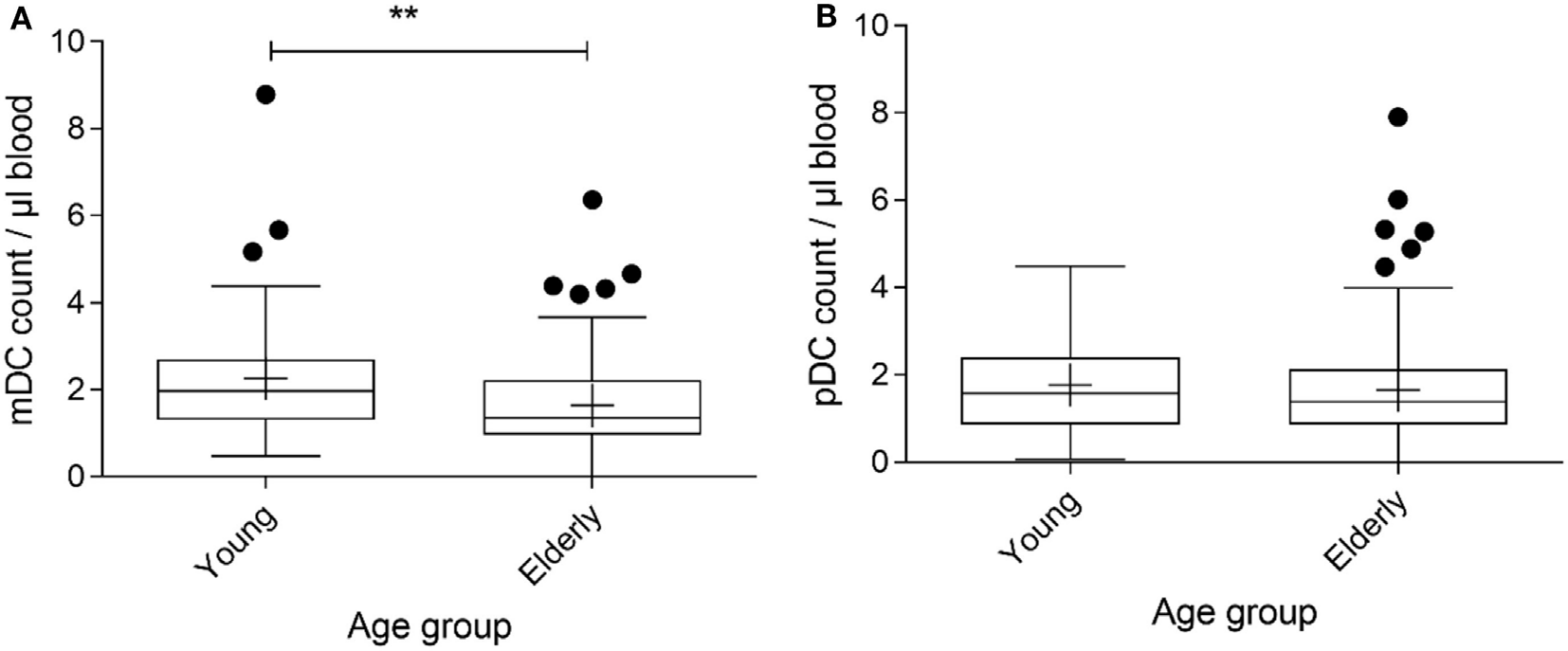
Myeloid DC (mDC) counts for the Im-AGE (young) and pre-intervention Nu-AGE (elderly) cohorts. Whole blood was stained with antibodies reactive with CD1c to identify mDCs, CD303 to identify pDCs, and to CD14 and CD19 to exclude CD14^+^ monocytes and CD19^+^ B cells, of which a high proportion express CD1c. *n* = 45 in the young cohort and *n* = 120 in the elderly cohort. Box and whisker plot of mDC **(A)** and pDC **(B)** counts for young and elderly subjects extending from the 25th to the 75th percentiles with the line through the box representing the median and plus (+) representing the mean value. Whiskers were determined using Tukey’s method using the 25th and 75th percentile plus 1.5 times the interquartile range (IQR) as the end of the whiskers. Dots represent individual participants where the values fell above the 25th or 75th quartile plus 1.5 times the IQR. Welch–Satterthwaite *t*-test on rank transformed data was used to determine the presence of differences between the young and elderly cohorts; significance assumed at *P* < 0.05, **P < 0.01, *P* = 0.0043 (mDCs) and *P* = 0.3108 (pDCs).

To obtain an accurate comparison of DC subset counts between the two age groups with minimal sample processing, whole blood was used with an antibody panel comprising the blood DC specific markers CD1c and CD303, excluding CD14 and CD19 positive monocytes/macrophages and B cells, respectively. Comparing the young to the elderly cohort for the mDC phenotype there was sufficient evidence to reject the null hypothesis of equal cell counts in each group. The young cohort had greater numbers of mDCs compared with the elderly cohort, with the difference estimated to be 0.4831 (0.1622, 0.7932; 95% confidence interval) with a corresponding significance level of *P* = 0.0043 (Figure 1A). However, when comparing the pDC phenotype between the young and elderly cohorts there was insufficient evidence to reject the null hypothesis of no difference between the cell counts in each group at the 5% significance level (*P* = 0.3108) (Figure 1B).

### DCs from Elderly Subjects Produce Less CXCL8 and Have Fewer DCs Producing IL-6^+^CXCL8^+^ and IL-1β^+^CXCL8^+^

Intracellular staining (ICS) is a robust method for observing cytokine production by individual cell populations within a complex multicellular sample that obviates the need for further manipulation to isolate cells of interest (36, 37). As the study samples were collected over the period of 2.5 years, it was not possible, or appropriate, to use fresh PBMCs for these experiments, therefore isolated PBMCs were cryopreserved before analysis.

**Figure 2:**
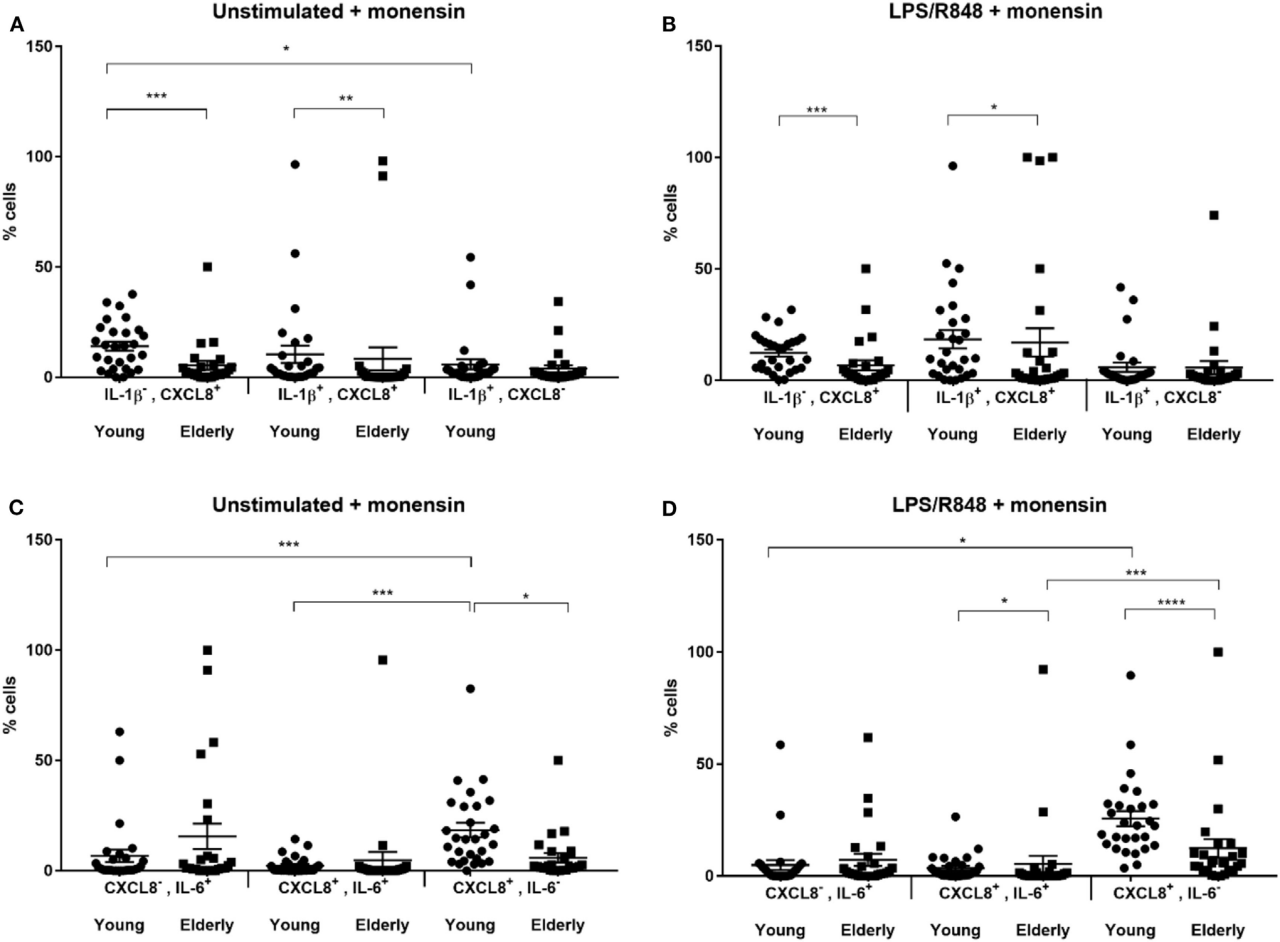
Scatter plots showing proportion of cytokine producing dendritic cells from young and elderly subjects. Peripheral blood mononuclear cells (PBMCs) were cultured *in vitro* for 3 h with tissue culture media alone **(A,C)** or in media containing lipopolysaccharide (LPS) and R848 **(B,D)** in the presence of 2 µM monensin. PBMC samples were subsequently surface stained with monoclonal antibodies against HLA-DR, CD1c, CD303, and CD304, CD14, CD16, CD19, and CD3, permeabilized and stained with anti-IL-1β, IL-6, and CXCL8 antibodies. *P < 0.05, **P < 0.01, ***P < 0.001, and ****P < 0.0001 indicate significance between age groups as measured by Mann–Whitney *U* tests and *post hoc* analyses, performed by one-way analysis of variance using the Kruskal–Wallis test with Dunn’s multiple comparisons *post hoc* test to identify any differences in proportions of the three cell types. Error bars show SEM and a horizontal line at the mean. CXCL8, C-X-C chemokine ligand 8; IL, interleukin; LPS, lipopolysaccharide, R848, resiquimod.

Comparing DCs from young and elderly subjects showed that the proportion of CXCL8^+^ and IL-1β^+^CXCL8^+^ double positive cells was significantly greater amongst DCs derived from young subjects compared with those from elderly subjects. This was apparent both with and without LPS, R848 stimulation (Figures 2A,B). The proportion of DCs secreting IL-1β was not significantly different between samples derived from young and elderly subjects. In addition, there were significantly more CXCL8^+^IL-6^+^ DCs in samples derived from young subjects compared with those from the elderly, but only after LPS, R848 stimulation (Figures 2C,D). The proportion of single positive DCs for CXCL8 (IL-6^−^CXCL8^+^) was significantly greater in samples from young subjects, while IL-6 single positive cells were not significantly different between the two groups; this was apparent for both non-stimulated and stimulated samples. Further analysis, by one-way ANOVA, identified significant differences in the proportion of non-stimulated IL-1β^−^CXCL8^+^ DCs and IL-1β^+^CXCL8^−^ cells, as well as between CXCL8^+^IL-6^+^ and CXCL8^−^IL-6^+^ compared with CXCL8^+^IL-6^−^ cells, and CXCL8^−^IL-6^+^ compared with CXCL8^+^IL-6^−^ DCs, in LPS and R848-stimulated samples from young subjects. While in the DCs from elderly subjects the only significant differences observed were between the proportion of CXCL8^+^IL-6^+^ compared with CXCL8^+^IL-6^−^ DCs in LPS and R848-stimulated samples.

Secretion of adipokines by PBMCs was significantly reduced in the elderly. Differences in secretion between baseline (non-stimulated) and post-stimulation with LPS and R848 for young subjects, as determined by paired *t*-tests (Table 2) was significantly different for MCP-1, IL-1β, IP-10, IL-10, CXCL8, IL-6, and TNF-α. However, differences in secretion by PBMCs from elderly subjects (Table 2) were only significant for IL-1β, CXCL8, IL-6, and TNF-α when comparing stimulated to non-stimulated samples. Comparison of cytokine secretion in samples from young to elderly subjects showed the levels of secretion were ~10-fold lower in samples from the elderly. To ensure accurate comparisons between samples any samples producing values below the level of detection were recorded as 0.0 pg/ml.

**Table 2:** Mean concentrations for each adipokine in cell culture supernatants from peripheral blood mononuclear cells with or without lipopolysaccharide, R848 stimulation from young and elderly subjects.

Adipokine	Age group		Media alone (pg/ml)	Stimulated (pg/ml)	*P* value
MCP-1	Young	Mean (SEM)	1,237 (279.00)	1,507 (315.90)	0.004
		Range	1.2–7,054	1.24–8,080	
	Elderly	Mean (SEM)	11.24 (3.37)	22.05 (8.40)	0.065 (ns)
		Range	0–57.54	0–199.90	
IL-1β	Young	Mean (SEM)	334.70 (191.00)	519.10 (149.40)	0.049
		Range	0–4,304	0–3,217	
	Elderly	Mean (SEM)	1.12 (0.53)	11.38 (3.36)	0.005
		Range	0–9.49	0–52.37	
IP-10	Young	Mean (SEM)	144.60 (96.79)	302.10 (130.60)	0.001
		Range	0–2,830	1.28–3,617	
	Elderly	Mean (SEM)	2.37 (0.83)	2.14 (0.89)	0.670 (ns)
		Range	0–17.14	0–21.72	
IL-10	Young	Mean (SEM)	18.89 (7.76)	40.73 (8.84)	0.006
		Range	0–178.90	0–149.90	
	Elderly	Mean (SEM)	0.08 (0.05)	0.13 (0.08)	0.166 (ns)
		Range	0–1.24	0–1.85	
CXCL8	Young	Mean (SEM)	2,987 (458.10)	4,998 (742.30)	0.003
		Range	5.36–8,231	5.12–15,430	
	Elderly	Mean (SEM)	115.30 (61.45)	346.10 (118.30)	0.007
		Range	0–1,610	0–2,422	
IL-6	Young	Mean (SEM)	529.70 (229.50)	1,392 (240.10)	0.001
		Range	0–4,880	0–4,470	
	Elderly	Mean (SEM)	2.34 (2.06)	15.43 (7.64)	0.034
		Range	0–53.43	0–187.40	
Resistin	Young	Mean (SEM)	169.40 (42.51)	140.90 (40.04)	0.054
		Range	0–998.40	1.43–991.40	
	Elderly	Mean (SEM)	121.60 (51.93)	136.40 (54.34)	0.264 (ns)
		Range	0–1,028	0–869.90	
TNF-α	Young	Mean (SEM)	81.06 (49.42)	2,782 (459.60)	<0.0001
		Range	0–1,207	0.9–7,550	
	Elderly	Mean (SEM)	0.77 (0.59)	48.27 (16.41)	0.008
		Range	0–15.42	0–350.90	

*CXCL8, C-X-C chemokine ligand 8; IL, interleukin; IP, interferon gamma inducible protein 10; ns, not statistically significant; MCP-1, monocyte chemoattractant protein-1; SEM, standard error of the mean; TNF-α, tumor necrosis factor alpha*.

Comparisons of the CFB after LPS and R848 stimulation between young and elderly subjects showed that the PBMCs from young subjects secreted higher concentrations of MCP-1, TNF-α, CXCL8, IL-1β, and IL-6 compared with those from elderly subjects, all of which were significantly greater (*P* < 0.05) (Table 2). By contrast, resistin secretion decreased after stimulation of the PBMCs from the young subjects (mean of 29 donors), while secretion from PBMCs derived from elderly subjects was statistically higher compared with the young subjects (*P* = 0.028). Change in levels of adiponectin after LPS, R848 stimulation were not significantly different between young and elderly subjects (*P* = 0.814; data not shown).

To ensure that any changes observed between groups were independent of plate-to-plate variability, the absolute concentrations for each serial dilution of the standards, which were run on every plate, were compared. Concentrations for all dilutions of the standard were comparable between all plates and all corresponded to the expected concentration, additionally, the coefficient of variance for each analyte on each plate were all within acceptable limits of less than 5%, highlighting the precision of each dataset (data not shown). All *R*^2^ values were ≥0.998.

In summary, we observed a significant reduction in numbers of mDCs within peripheral blood, in addition to significantly reduced secretion of pro-inflammatory cytokines (IL-1β, CXCL8, IL-6, TNF-α, and MCP-1), and elevated secretion of resistin upon TLR stimulation by DCs and PBMCs, respectively. The potential for MED-dietary intervention to impact on these findings was investigated in elderly individuals over a 1-year period.

### Demographics of MED-Diet Intervention Participants

Both MED diet and control groups had lower mean BMI (−0.3 kg/m^2^) and weight (−0.5 kg) values after the year intervention; however, comparison of the CFB between the two groups was not significantly different for either variable. Similarly, height, waist circumference, and frailty status changes from baseline were comparable with no significant difference observed between the groups at post-intervention (Table 3). Ten participants dropped out of the study; however, the gender ratios of the two groups remained similar at post-intervention, with no significant difference observed between the groups at post-intervention (Table 3). The results of DXA scans at baseline and 1-year post-intervention show that there were minimal changes to the mean body weight, fat mass, lean mass, bone mineral content (BMC), soft tissue and BMD, while the fat mass and regional fat mass percentages were reduced at post-intervention but minimally (Table S2 in Supplementary Material). The mean *T* scores were similar but the upper value of the range was much smaller, indicating deterioration in some subjects (Table S2 in Supplementary Material). Plasma lipid analysis shows that while all subjects had high total and LDL cholesterol, and normal HDL and triglyceride concentrations, these concentrations were unaffected in both dietary groups (Figure S2 in Supplementary Material).

**Table 3:** Characteristics of study participants at pre-compared to post-intervention.

		Pre-intervention		Post-intervention		Mean CFB in control group	Mean CFB in MED group	*P* values (control v MED diet)
		Control group (*n* = 57)	Mediterranean (MED)-diet group (*n* = 65)	Control group (*n* = 54)	MED-diet group (*n* = 61)			
Age (years)	Mean (SD)	71.0 (4.1)	69.8 (4.2)	71.6 (3.8)	70.8 (4.2)	0.6	1.0	0.55
	Range	65–79	65–79	66–80	66–80			

Gender	% Female	61	60	61	57	0	−3	–

BMI (kg/m^2^)	Mean (SD)	26.6 (3.3)	26.7 (4.5)	26.3 (3.6)	26.4 (4.6)	−0.3	−0.3	0.99
	Range	20.0–37.4	18.5–43.2	18.9–37.9	18.2–45.8			

Weight (kg)	Mean (SD)	73.2 (12.5)	73.4 (15.1)	72.7 (12.6)	72.9 (14.7)	−0.5	−0.5	0.40
	Range	52.5–108.9	49.5–128.5	52.0–101.0	49.7–118.7			

Height (cm)	Mean (SD)	165.7 (9.3)	165.6 (9.1)	166.1 (9.3)	165.8 (9.0)	0.4	0.2	0.26
	Range	148.6–187.4	145.6–188.2	149.6–186.5	146.6–187.1			

Waist circumference (cm)	Mean (SD)	91.5 (11.1)	91.3 (12.5)	91.2 (11.7)	91.5 (12.6)	−0.3	0.2	0.96
	Range	72.0–122.0	63.5–134.8	69.1–127.9	65.0–126.7			

Frailty status (*n*)	Non-frail	12	14	9	9	1	12	0.62
	Pre frail	44	50	42	54	−4	−7	
	Frail	2	0	0	0	−0.04	0	

*P values determined by paired t-tests*.

*CFB, change from baseline; MED-diet, mediterranean diet; SD, standard deviation*.

### Diet Had No Effect on DC Subset Distribution or Cytokine Production in Elderly Subjects

**Figure 3:**
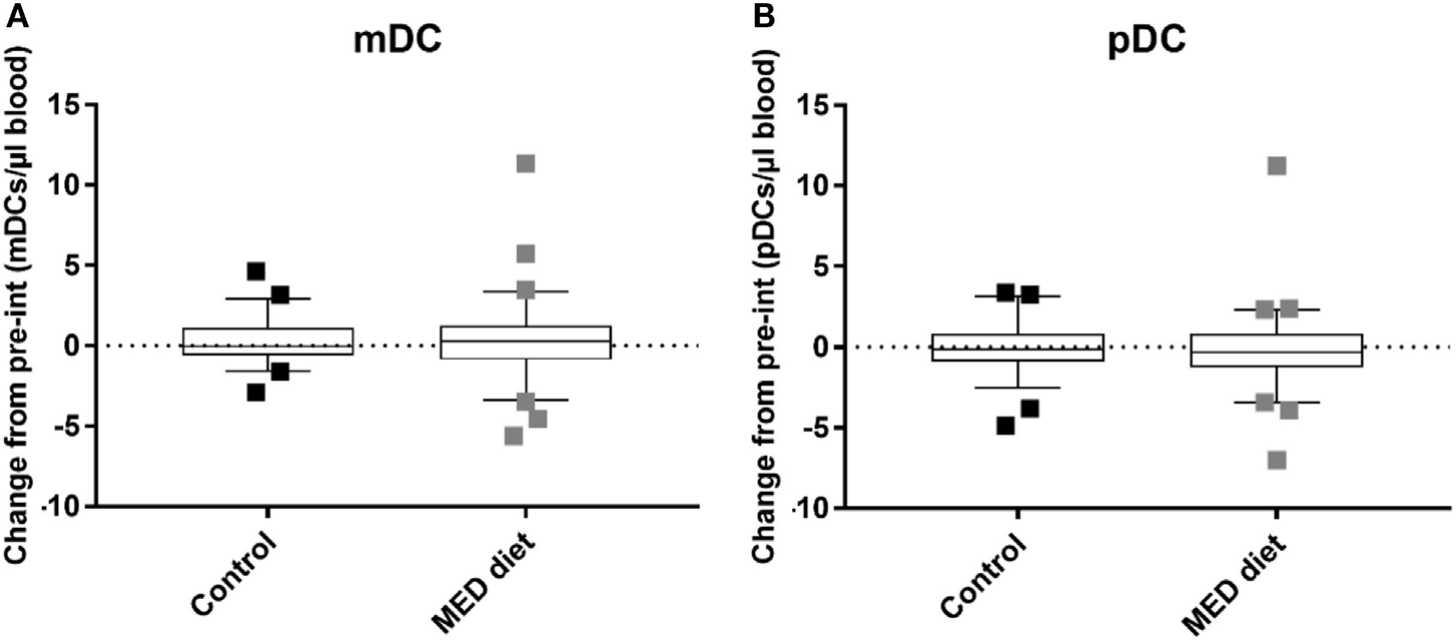
Myeloid DC (mDC) and plasmacytoid DC (pDC) counts after dietary intervention for control and MED diet study groups. Whole blood from control and dietary intervention groups was stained with anti-CD1c to identify mDCs, CD303 to identify pDCs, and anti-CD14 and CD19 to exclude CD14^+^ monocytes and CD19^+^ B cells, of which a high proportion express CD1c. The data show box and whisker plots of change in mDC **(A)** and pDC **(B)** counts from pre- to post-intervention in elderly subjects extending from the 25th to the 75th percentiles, with the horizontal line representing the median. Whiskers were determined using Tukey’s method using the 25th and 75th percentile plus 1.5 times the interquartile range (IQR) as the end of the whiskers. Squares represent individual participants where the values fell above the 25th or 75th quartile plus 1.5 times the IQR. A Mann–Whitney *U* test was used to determine the presence of differences between the control and dietary intervention cohorts. *n* = 58, Control group, *n* = 62, Mediterranean (MED)-diet group, significance assumed at *P < 0.05, **P < 0.01, *P* = 0.9104 (mDCs) and *P* = 0.3553 (pDCs).

No effect was observed in numbers of either mDC (Figure 3A) or pDC (Figure 3B) subsets, which remained consistent over the dietary intervention for both groups. There was therefore insufficient evidence to reject the null hypothesis of no difference between the cell counts in each group at the 5% significance level.

**Figure 4:**
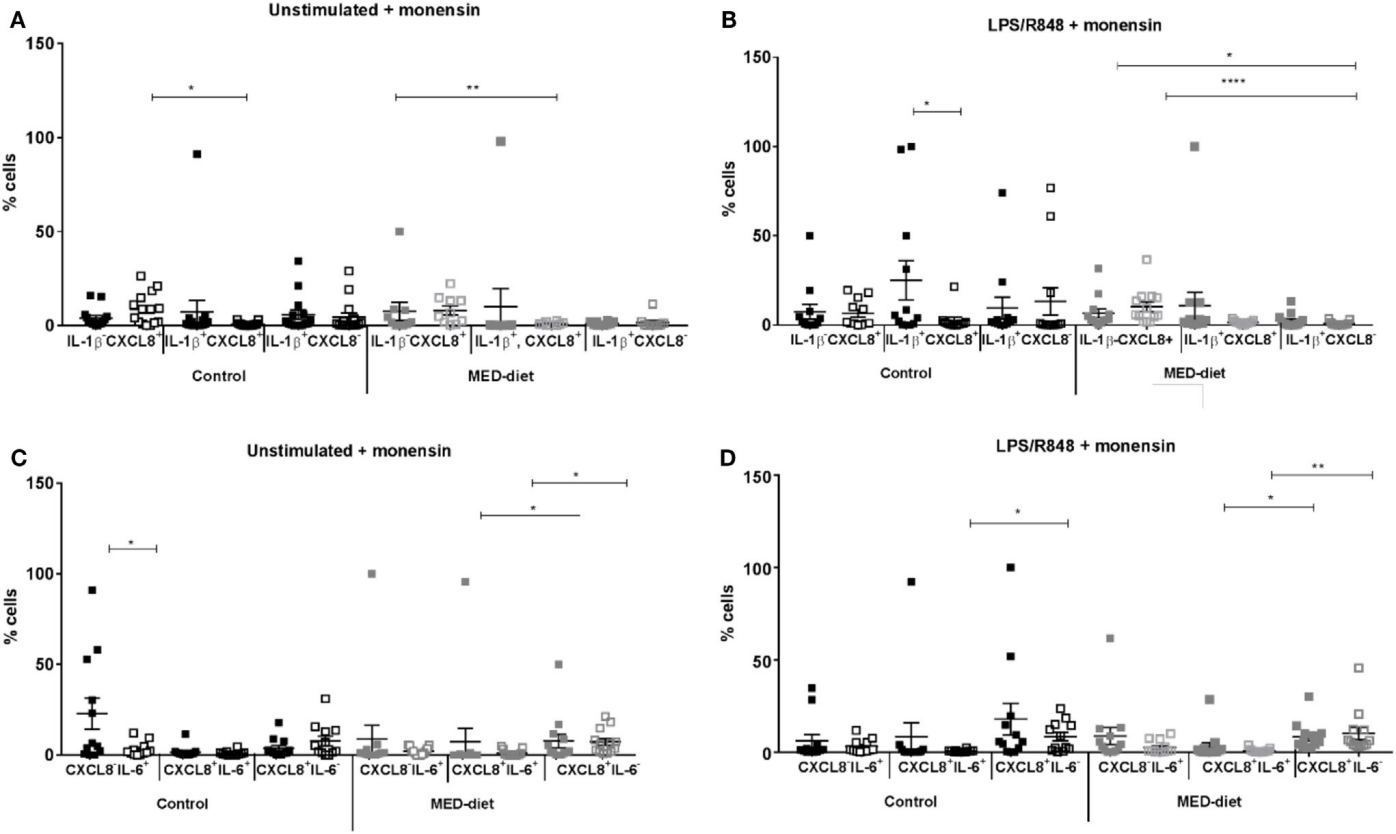
Cytokine producing dendritic cells from elderly subjects at pre- and post-dietary intervention. Peripheral blood mononuclear cells from control subjects and Mediterranean (MED)-diet intervention subjects were cultured for 3 h in complete tissue culture media alone **(A,C)**, or in media containing lipopolysaccharide (LPS) and R848 **(B,D)** in the presence of 2 µM monensin. Samples were subsequently surface stained with anti-HLA-DR, CD1c, CD303, and CD304, CD14, CD16, CD19, and CD3 antibodies, permeabilized and stained with anti-IL-1β IL-6 and CXCL8 antibodies. Squares represent values for each individual subject with filled squares (■) representing pre-intervention values and open squares (□) representing post-intervention values. Significance assumed at *P* < 0.05; *P < 0.05, **P < 0.01, ***P < 0.001, and ****P < 0.0001 indicates significance between pre- and post-dietary intervention as measured by Wilcoxon matched pairs signed rank test, and *post hoc* analyses, performed by one-way analysis of variance using the Kruskal–Wallis test with Dunn’s multiple comparisons *post hoc* test to identify any differences in proportions of the three cell types. Error bars show SEM and a horizontal line at the mean. CXCL8, C-X-C chemokine ligand 8; IL, interleukin; LPS, lipopolysaccharide, R848, resiquimod.

Significant reductions in IL-1β^+^CXCL8^+^ DCs were observed between pre- and post-intervention in the control group with LPS, R848 stimulation (Figure 4B; *P* = 0.0264). Also, a significant reduction was observed in CXCL8^−^IL-6^+^ DCs in non-stimulated samples from the control group (Figure 4C; *P* = 0.0269). Further analysis (by one-way ANOVA) showed the numbers of IL-1β^−^CXCL8^+^ DCs were significantly greater than the number of IL-1β^+^CXCL8^+^ DCs at post-intervention in non-stimulated samples (Figure 4A; *P* = 0.0146) and the numbers of CXCL8^+^IL-6^+^ DCs at post-intervention were significantly reduced compared with CXCL8^+^IL-6^−^ DCs at post-intervention (Figure 4D; *P* = 0.0380), after LPS, R848 stimulation.

In the MED-diet group, no significant differences were observed on comparison of the same cell type from pre- to post-intervention in LPS, R848-stimulated or non-stimulated samples (*P* > 0.05). After further analyses significant reductions were observed in CXCL8^+^IL-6^+^ compared with CXCL8^+^IL-6^−^ DCs both at pre- (*P* = 0.0118) and post-intervention (*P* = 0.0154) for unstimulated samples. The same was also observed in LPS, R848-stimulated samples (*P* = 0.0051 and *P* = 0.0008 respectively), in addition significantly greater numbers of IL-1β^−^CXCL8^+^ DCs at pre- and post-intervention compared with IL-1β^+^CXCL8^−^ DCs at post-intervention (*P* = 0.0105 and <0.0001; respectively).

### The MED-Diet Reduced Resistin Production by PBMCs

There were no significant differences in secretion of any of the cytokines analyzed in the control group by non-stimulated or LPS, R848-stimulated PBMCs at baseline (Table 4) as determined by paired *t*-tests. While in the MED-diet group LPS, R848 stimulation induced significant increases in IL-1β, CXCL8, and TNF-α secretion (Table 5). The absolute concentrations recorded for the two groups at baseline were similarly low for Adipsin, MCP-1, IP-10, IL-6 (Table 5), and IFN-γ (data not shown).

**Table 4:** Absolute concentrations of cytokines in peripheral blood mononuclear cell culture supernatant, from elderly subjects in the control dietary intervention group, at pre- and post-intervention.

Adipokine	Study time point		Media alone	Stimulated	*P* values
Adipsin	Pre-int	Mean (SEM)	21.28 (9.66)	25.13 (10.8)	0.551 (ns)
		Range	0–98.29	0–91.65	
	Post-int	Mean (SEM)	96.99 (62.22)	89.06 (58.75)	0.076 (ns)
		Range	0–710.70	0–669.50	
MCP-1	Pre-int	Mean (SEM)	4.50 (3.02)	6.81 (4.69)	0.205 (ns)
		Range	0–34.13	0–52.88	
	Post-int	Mean (SEM)	16.48 (6.70)	29.77 (12.53)	0.177 (ns)
		Range	0–71.79	1.11–121	
IL-1β	Pre-int	Mean (SEM)	1.83 (1.01)	4.58 (2.49)	0.275 (ns)
		Range	0–9.49	0–25.03	
	Post-int	Mean (SEM)	25.69 (17.19)	29.22 (17.06)	0.026
		Range	0–153.70	0–158.90	
IP-10	Pre-int	Mean (SEM)	4.59 (1.75)	3.29 (1.93)	0.227 (ns)
		Range	0–17.14	0–21.72	
	Post-int	Mean (SEM)	1.35 (0.59)	1.32 (0.60)	0.963 (ns)
		Range	0–5.17	0–5.73	
CXCL8	Pre-int	Mean (SEM)	32.84 (19.74)	104.4 (52.48)	0.065 (ns)
		Range	0–220.70	0–580.50	
	Post-int	Mean (SEM)	318.5 (185.80)	552.10 (169.80)	0.070
		Range	3.48–1,751	4.87–1,668	
IL-6	Pre-int	Mean (SEM)	0.50 (0.50)	3.90 (2.83)	0.185 (ns)
		Range	0–5.48	0–29.38	
	Post-int	Mean (SEM)	32.38 (22.26)	33.60 (19.39)	0.761 (ns)
		Range	0–214.30	0–181.10	
Resistin	Pre-int	Mean (SEM)	164.90 (100.30)	183.70 (100.5)	0.531 (ns)
		Range	0–1,028	0–869.90	
	Post-int	Mean (SEM)	309.10 (124.00)	285.10 (120.70)	0.124 (ns)
		Range	0–1,245	0–1,246	
TNF-α	Pre-int	Mean (SEM)	0.09 (0.09)	16.68 (7.67)	0.054 (ns)
		Range	0–0.96	0–75.91	
	Post-int	Mean (SEM)	2.95 (2.01)	53.26 (16.63)	0.016
		Range	0–18.85	0–151	

*CXCL8, C-X-C chemokine ligand 8; IL, interleukin; IP-10, interferon gamma inducible protein 10; LPS, lipopolysaccharide; ns, not statistically significant; MCP-1, monocyte chemoattractant protein-1; R848, resiquimod; SEM, standard error of the mean; TNF-α, tumor necrosis factor alpha*.

**Table 5:** Absolute concentrations of cytokines in peripheral blood mononuclear cell culture supernatant, from elderly subjects in the Mediterranean-dietary intervention group, at pre- and post-intervention.

Adipokine	Study time point		Media alone	Stimulated	*P* values
Adipsin	Pre-int	Mean (SEM)	3.83 (2.12)	4.74 (3.89)	0.743 (ns)
		Range	0–23.14	0–57.72	
	Post-int	Mean (SEM)	103.40 (48.23)	92.73 (48.59)	0.132 (ns)
		Range	0–661.30	0–651.00	
MCP-1	Pre-int	Mean (SEM)	16.19 (5.12)	33.22 (13.63)	0.093 (ns)
		Range	0–57.54	0–199.90	
	Post-int	Mean (SEM)	199.80 (183.30)	179.20 (166.00)	0.264 (ns)
		Range	0–2,398	0–2,170	
IL-1β	Pre-int	Mean (SEM)	0.59 (0.54)	16.37 (5.25)	0.009
		Range	0–8.17	0–52.37	
	Post-int	Mean (SEM)	41.26 (30.94)	16.57 (11.54)	0.447 (ns)
		Range	0–401.60	0–152.20	
IP-10	Pre-int	Mean (SEM)	0.73 (0.33)	1.30 (0.61)	0.225 (ns)
		Range	0–4.28	0–7.67	
	Post-int	Mean (SEM)	30.97 (28.74)	9.89 (7.62)	0.338 (ns)
		Range	0–375.70	0–101.00	
CXCL8	Pre-int	Mean (SEM)	175.80 (104.20)	523.40 (191.30)	0.018
		Range	0–1,610	7.07–2,422	
	Post-int	Mean (SEM)	810.60 (395.90)	961.10 (480.10)	0.226 (ns)
		Range	0–4,738	17.27–6,130	
IL-6	Pre-int	Mean (SEM)	3.69 (3.56)	23.89 (12.82)	0.056 (ns)
		Range	0–53.43	0–187.40	
	Post-int	Mean (SEM)	218.60 (208.80)	15.71 (6.24)	0.342 (ns)
		Range	0–2,723	0–64.81	
Resistin	Pre-int	Mean (SEM)	89.80 (53.89)	101.70 (60.39)	0.204 (ns)
		Range	0–747.20	0–790.70	
	Post-int	Mean (SEM)	304.60 (110.40)	251.10 (101.80)	0.049
		Range	14.03–1,320	12.98–1,262	
TNF-α	Pre-int	Mean (SEM)	1.28 (1.02)	71.44 (26.68)	0.021
		Range	0–15.42	0–350.90	
	Post-int	Mean (SEM)	394.20 (393.10)	18.35 (6.57)	0.360 (ns)
		Range	0–5,111	0–64.89	

*CXCL8, C-X-C chemokine ligand 8; IL, interleukin; IP-10, interferon gamma inducible protein 10 LPS, lipopolysaccharide; ns, not statistically significant; MCP-1, monocyte chemoattractant protein-1; R848, resiquimod; SEM, standard error of the mean; TNF-α, tumor necrosis factor alpha*.

At post-intervention (Table 4) in the control group the secretion of IL-1β and TNF-α were significantly increased after LPS, R848 stimulation. The concentration of resistin was significantly reduced post-MED dietary intervention (Table 5). All other analytes remained the same, with no significant differences observed between non-stimulated and LPS, R848-stimulated PBMC samples.

**Figure 5:**
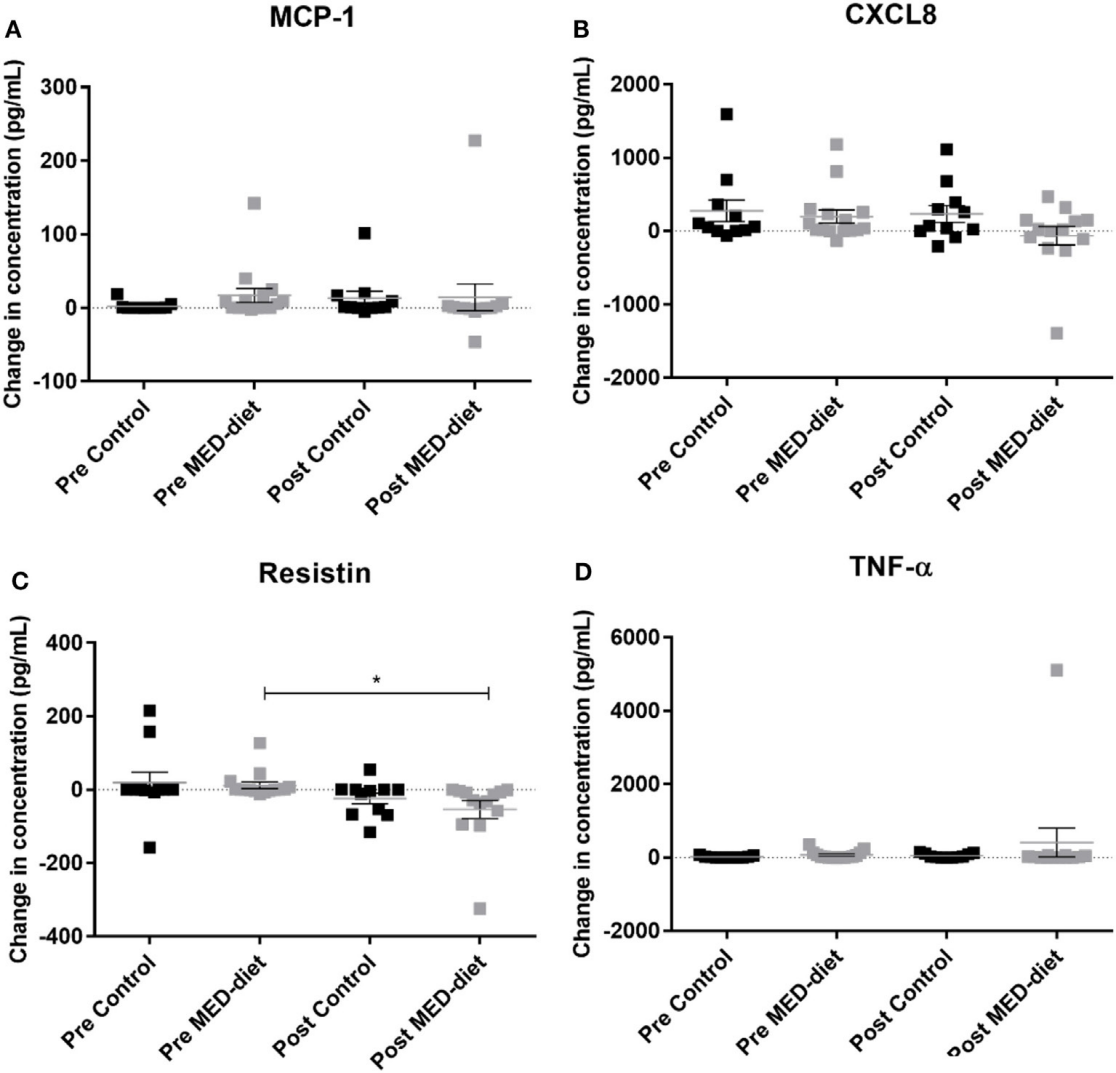
Change in cytokine production by peripheral blood mononuclear cells (PBMCs) in response to lipopolysaccharide (LPS) and R848 stimulation in pre- versus post-dietary intervention. PBMCs from control subjects and Mediterranean (MED)-diet intervention subjects were cultured for 3 h in complete tissue culture media alone, or in media containing LPS and R848 in the presence of 2 µM monensin. PBMC culture supernatants were analyzed by multiplex bead based immunoassay to determine absolute concentrations of MCP-1 **(A)**, CXCL8 **(B)**, resistin **(C)** and TNF-α **(D)** in the PBMC samples. Concentrations (pg/ml) in non-stimulated samples were subtracted from stimulated samples to give a change in concentration as a result of the stimulus. Squares represent individual values for change in secretion of each cytokine by PBMCs, error bars represent the SEM. Determination of significant differences between pre- and post-intervention by paired *t*-tests, significance assumed at *P < 0.05, **P < 0.01, ***P < 0.001. CXCL8, C-X-C chemokine ligand 8, MCP-1, monocyte chemoattractant protein-1 ns; not statistically significant; TNF-α, tumor necrosis factor alpha.

**Figure 6:**
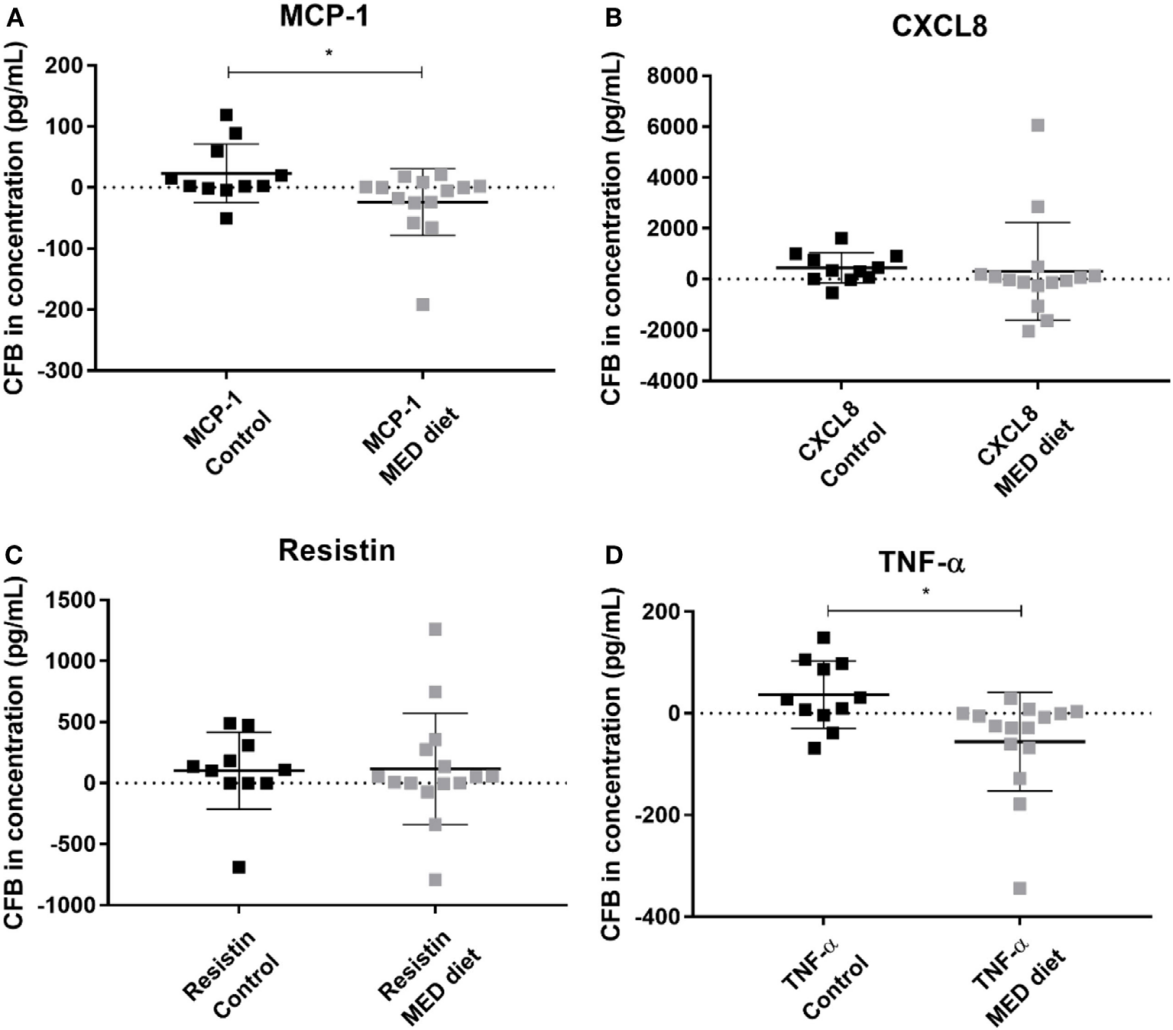
Change from baseline (CFB) in cytokine production by PBMCs in response to lipopolysaccharide (LPS) and R848 stimulation after 1 year of control and MED-diet intervention. PBMCs from control subjects and MED-diet intervention subjects were cultured for 3 h in complete tissue culture media containing LPS and R848 in the presence of 2 µM monensin. PBMC culture supernatants were analyzed by multiplex bead based immunoassay to determine absolute concentrations of MCP-1 **(A)**, CXCL8 **(B)**, resistin **(C)** and TNF-α **(D)** in the PBMC samples. Concentrations (pg/mL) of each cytokine at pre-intervention were subtracted from the post-intervention values to give a CFB value. CFB in the control and MED-diet groups was compared using a Mann–Whitney *U* test, with significance assumed at *P < 0.05. CFB, change from baseline; CXCL8, C–X–C chemokine ligand 8; MCP-1, monocyte chemoattractant protein-1; MED: Mediterranean; PBMC, peripheral blood mononuclear cell; TNF-α, tumor necrosis factor alpha.

Comparison of PBMC cytokine secretion from pre- to post-intervention showed that all analytes investigated, except for resistin, were not significantly affected by dietary intervention (Figures 5A–D). Comparing changes in resistin concentration, between LPS, R848-stimulated pre- and post-intervention for each group revealed that while the resistin levels were not significantly different between the pre- and post-intervention sample in the control group, they were significantly reduced at post-intervention in the MED-diet group (Figure 5C). However, upon comparison of the CFB between the control and MED groups, it was apparent that the change in resistin secretion, after LPS, R848 stimulation, was not significantly different between the two groups (Figure 6C). Similarly, CFB in CXCL8 was not significantly different between the control and MED-diet groups (Figure 6B), while CFB in MCP-1 and TNF-α were significantly different between control and MED-diet subjects (Figures 6A,D).

### MED-Diet Scores Were Not Different between Control and MED-Diet Study Participants but MED-Diet Subjects Consumed More Phenolic Compounds

To assess compliance self-reported 7DDs were kept at baseline and post-intervention by the study participants, which allowed MED-diet scores to be calculated for each participant, pre- and post-intervention, according to the method described by Sofi et al. (38). The mean MED-diet score for the control group was 5.8, and for the MED-diet group was 5.5 at pre-intervention. In both groups, the mean scores decreased at post-intervention, to 4.7 and 5.1, respectively (Table S3 in Supplementary Material). When comparing mean change in MED-diet scores from baseline for the two groups, the control group decreased, on average by one point, while there was little change in the MED-diet group. Despite the significant difference observed between the mean change in MED-diet scores between the two study groups (Figure S2 in Supplementary Material), there was no evidence of an association between score and group allocation since the MED-diet score did not increase for the subjects in the MED-diet group.

To ascertain a biologically determined indication of study participants’ compliance, the levels of the olive oil metabolite, hydroxytyrsol sulfate (HTS), in 24-h urine samples collected from a subset of the Nu-AGE participants were analyzed, according to ion fragmentation by HPLC coupled with tandem MS. The data show, surprisingly, that HTS was present in all samples at baseline, with a minimum concentration of 90 ng/ml. Upon grouping the samples according to study group allocation, control or intervention, the MED-diet group showed a greater increase from baseline in concentration of HTS, with 11 out of the 17 participants showing an increase as opposed to a decrease from baseline levels (data not shown). When HTS concentrations were compared using an unpaired *t*-test (Figure S3 in Supplementary Material) there was strong evidence that HTS concentrations differed between the two groups (*P* < 0.05). In addition, the group with the higher levels of HTS was also the group which was found to consume significantly greater quantities of olive oil, determined when analyzing data from their 7DDs.

## Discussion

### Numbers of mDCs Are Reduced in the Elderly, while pDC Numbers Remain Similar to Young Subjects

Our finding that the reduction of mDCs but not pDCs with age is supported by Della Bella et al. (12) who also analyzed whole peripheral blood samples, in contrast to other studies using PBMCs that found reductions in pDCs (8, 10, 39). This discrepancy could be due to sample manipulation as suggested by Gerrits et al. (40) who compared fresh blood samples to Ficoll-isolated fresh and cryopreserved PBMCs using anti-CD1c, anti-CD303, anti-CD19, and anti-CD14 antibodies and found that PBMC isolation resulted in an approximate threefold increase in mDCs and pDCs when compared with fresh blood, and cryopreservation produced a fivefold increase. A previous study using very similar methodology to that described here produced opposing results (9). A possible reason for this may be that identification of CD1c and CD303 expressing blood DCs by Pérez-Cabezas et al. (9) did not exclude CD14, CD19, or CD20 and may have therefore overestimated mDCs numbers; B cells also express CD1c (41) and CD14^+^ monocytes present in PBMCs after CD19 depletion express CD1c (42). As we only show reductions in mDC counts it seems unlikely that this is a result of defective DC precursors with increased age, since both pDCs and mDCs are derived from macrophage and DC precursors and differentiate into common DC precursors (43). However, with regard to the reduction in mDCs, the finding of increased numbers of CD14^+^ monocytes and decreased numbers of CD34^+^ precursors (hematopoietic stem cells) alongside the reduction in mDCs by Della Bella et al. (12) implies there may be an age-associated impact on the differentiation of these cells, preventing DC differentiation. However, as this was not investigated here these findings cannot be confirmed in the Norfolk cohort.

### DCs from Elderly Subjects Produce Less CXCL8 and Have Fewer DCs Producing IL-6^+^ CXCL8^+^ and IL-1β^+^ CXCL8^+^

The proportions of DCs secreting CXCL8 alone or in combination with IL-1β or IL-6 are significantly reduced with age after stimulation with LPS and R848. The highest proportion of cytokine secretion by DCs derived from both young and elderly donors is seen for CXCL8^+^IL-6^−^ producing DCs. The results from non-stimulated and stimulated DCs are similar, except no statistically significant difference is observed between CXCL8^+^IL-6^+^ DCs from young and elderly subjects. A similar study using frozen PBMCs as a source of DCs for ICS (44) showed that, after 6 h stimulation with 0.5 µg/ml of LPS, DCs from elderly subjects produced significantly greater amounts of IL-6 (and TNF-α) than the non-stimulated cells with the increase being small but statistically significant. In this study Janssen et al. (44) recruited cytomegalovirus (CMV) seropositive young subjects, to exclude CMV seropositivity as a confounding factor when comparing results with elderly individuals; this could explain the differing results observed between this and our study.

We acknowledge that the differing results could also be a result of the length of time that the PBMCs were stimulated since in addition to use of a different source of DCs, increases in secretion of IL-6 from MoDCs have been observed with LPS stimulation (45, 46) for 24 and 48 h, respectively. The kinetics of DC cytokine secretion has been investigated to show that while DCs “exhaust” their capacity to produce IL-12 after long stimulation periods (such as 24 and 48 h), capacity to secrete IL-6 remained intact, even at 48 h (47), and could be detected after just 3–4 h. The reduction in mDC numbers observed in the elderly cohort compared with the young cohort, may also be a contributing factor to the reduction in cytokine secretion observed in this study.

#### Secretion of Adipokines by PBMCs Is Significantly Reduced in the Elderly

We found significant reductions in MCP-1, TNF-α, CXCL8, IL-6, and IL-1β in PBMCs from elderly subjects. A similar study comparing cell culture supernatants from MoDCs stimulated with LPS (for 20–24 h) found significantly increased concentrations of IL-6 and TNF-α with single-stranded RNA stimulation also significantly increasing TNF-α secretion in the MoDCs from elderly subjects (46). However, mDCs and pDCs isolated from PBMCs of young and elderly subjects and stimulated with poly I:C and influenza virus, respectively, found that pDCs secreted less IFN-α, IL-6, and TNF-α compared with young controls, while mDCs secreted comparable levels of cytokines (8). In addition, after 24 h LPS stimulation of PBMCs, but not whole blood, IL-6, CXCL8, and IL-1β secretion decreased (48), and in whole blood supernatants IL-1β and TNF-α secretion declined in samples from elderly compared with young subjects (49). Therefore, the source of cell samples may again be a determining factor in whether cytokine secretion increases or decreases upon TLR stimulation.

### Resistin Secretion by PBMCs Increases with Age

The present finding of significantly increased resistin production, by LPS and R848 stimulated PBMCs from elderly compared to young subjects, after LPS and R848 stimulation is interesting since inflammatory events can induce resistin production (50). Resistin, in humans, is expressed predominantly in the bone marrow (BM) but is also present in circulating blood (51) and PBMCs are an important source (52). Our finding of increased resistin production by PBMCs from elderly subjects implies a potential underlying inflammatory state or inflammaging. LPS has been shown to increase resistin gene expression from human PBMCs while these data are only based on samples from three healthy volunteers, the increases in resistin were substantial and consistent between all volunteers (53); a more quantitative measure, such as a cytokine bead array, would aid in the explanation. Resistin has also been shown to suppress the ability of MoDCs to secrete IL-6, TNF-α, and IL-12 (p40) after incubation with lipoteichoic acid (LTA) from *Staphylococcus aureus* (54). This suggests that the observed decreases in IL-6 and TNF-α, and possibly the other cytokines in this study could have been due to the elevated secretion of resistin in the elderly subjects. Contrary to these findings, previous research has shown that plasma concentrations of resistin in a healthy population of over 250 subjects did not differ with donor age (55). This may have been because the recruited cohort were healthy as previous observations of higher serum levels of resistin were also associated with increasing risk of CVD events (56). Resistin is also influenced by insulin resistance and adiposity, which was demonstrated upon comparison of offspring of non-long-lived individuals and centenarians (57).

When considering the two cohorts of volunteers recruited for this study there were no significant differences in mean BMI or weight of the individuals. After identifying individuals exhibiting increases in resistin PBMC secretion levels, BMI or weight were not consistently high for these individuals. In fact, out of the four individuals identified only one had a high BMI (pre-obese) (27.1 kg/m^2^), the rest were within the normal range (18.50–24.99 kg/m^2^) (58) and the highest concentration recorded was by an elderly individual with a healthy BMI of 23.0 kg/m^2^. Similarly, the DXA scan results from the elderly subjects also show that the individuals with the highest resistin concentrations had a range of fat mass percentages from 19.2 to 39.8% and fat/lean mass ratios of 0.2 to 0.7. This implies that while adiposity is commonly attributed with elevated resistin levels this may not be the cause of the observed increase in resistin in this study. Although increases in body fat and reductions in fat free mass occur with age and differences were only seen in resistin concentration when subjects were grouped by body fat content as opposed to BMI (55).

The impact of this finding, in addition to the current understanding of resistin in terms of healthy aging, implies that preservation of insulin sensitivity with increased age may be of importance to preserve health of the elderly. Resistin suppresses secretion of IL-6, IL-12, and TNF-α by LTA-stimulated MoDCs (54) with IL-6 and a proliferation-inducing ligand being important for maintenance of long-lived plasma cells, which decrease with age (59). Therefore, the age-associated reductions observed in cytokine secretion (IL-6, CXCL8, and IL-1β) but increases in the adipokine, resistin, could be due to an inflammatory state, typical of inflammaging.

### Diet Has No Effect on DC Subset Distribution in Elderly Subjects

Our observation that the MED diet has no effect on mDC or pDC numbers contrasts with the finding of Rehman et al. (60) that showed inhibited production of both murine derived BM DCs and human PBMC derived MoDCs upon blocking of FA synthesis using an inhibitor of acetyl CoA carboxylase (TOFA). However, the consistency in DC numbers from both subsets at 1-year post-intervention may be a positive finding as the numbers of mDCs or pDCs do not decrease further with an additional year of age. This has not been explored in previous studies that relied on one-off blood or PBMC samples from young and elderly subjects.

### The MED-Diet Changes DC Cytokine Secretion

The MED diet had no impact on DC cytokine secretion after 1-year intervention. However, significant differences were observed between pre- and post-MED-diet intervention between CXCL8^+^IL-6^+^ and CXCL8^+^IL-6^−^ DCs, IL-1β^−^CXCL8^+^ and IL-1β^+^CXCL8^−^ DCs, after LPS, R848 stimulation. While secretion of CXCL8 was seen to decrease with age, post-MED diet CXCL8 secretion increases, with significantly greater proportions of CXCL8^+^IL-6^−^ DCs compared with CXCL8^+^IL-6^+^ DCs, as well as greater proportions of IL-1β^−^CXCL8^+^ DCs compared with IL-1β^+^CXCL8^−^ DCs. These differences may be indicative of EPA and DHA (from consumption of oily fish) since these FAs diminish IL-6 secretion by LPS-stimulated MoDCs, with DHA treatment providing the most prominent effects (14). In addition, DHA decreases IL-1β secretion after palmitic acid induction (61), though these results were derived from a monocyte cell line, so cannot be directly compared with data from human blood derived DCs. Though the recent findings of Stelzner et al. (62) using LPS-stimulated MoDCs yield similar findings of palmitic acid induced increases in IL-1β and IL-6, although no effect of oleic acid, along with no effect of human consumption of DHA and EPA rich capsules on cytokine secretion by PBMCs isolated from these subjects (63). Therefore, DHA inhibition of DC activation *via* PPARγ and retinoic X receptors (14) could explain the present findings of reductions in secretion of IL-6, although current evidence is limited due to very few studies being carried out to date.

### The MED Diet Reduces Resistin Production

Resistin concentration, after stimulation with LPS, R848, was significantly reduced from pre-intervention after a 1-year intervention of a MED diet. This finding could be related to weight loss of the participants, as a recent study shows resistin levels positively correlate with BMI as well as abdominal visceral and subcutaneous fat volume and mass, determined by computer tomography (56). However, a weight loss study showed no effect on plasma resistin levels after a short term, 4-week reduced calorie diet, resulting in a mean weight loss of 3.4 kg (64). In addition, another group showed no association between plasma resistin levels and intra-abdominal fat levels, insulin sensitivity nor MetS, and only a weak association with BMI (65). The relationship between the adiposity and resistin levels is likely, therefore, to be complicated. The weight loss achieved by the participants in this study was small (−0.5 kg) and was not significantly different between the two study groups, and therefore may not be causal in reducing resistin levels. This is especially apparent as the control group did not have a significant reduction in resistin at post-intervention. However, it should be noted that the CFB in resistin secretion was not significantly different between the two study groups.

We show that the MED diet consuming study participants consumed increased amounts of phenolic compounds, most likely from olive oil, consistent with elevated urinary HTS concentrations in this group. This suggests that the subjects were compliant in terms of increasing consumption of phenolic-rich foods, so the effects observed may be a consequence of altered dietary intake. In considering FA intake calculated form the self-report 7DD it is apparent that the MED-diet group consumed significantly less saturated fat (*P* < 0.0001) compared with the control group while the MUFA and PUFA intakes were not significantly different between the groups (*P* = 0.063 and 0.902, respectively; data not shown). The MUFA intake is decreased compared with baseline for both groups in this study, suggesting the effects observed may be attributed to changes in the SFA:MUFA or SFA:PUFA ratio, or simply the reduced intake of SFA.

Previous studies show that IL-6, TNF-α, and IL-1β can induce secretion of resistin and that the insulin sensitizer, rosiglitazone, can neutralize these cytokines by activating PPARγ (66), suggesting that the presence of low-grade chronic inflammation may enhance secretion of resistin in the elderly subjects in this study. Since in this study levels of IL-6, TNF-α, and IL-1β were already low in the elderly subjects, and no significant reduction was observed after 1-year MED-diet intervention in any of these cytokines, the diet could potentially induce a reduction in resistin secretion *via* activation of PPARγ. However, the lack of a significant difference between the control and MED-diet groups for change in resistin production from baseline should be noted, while a significant reduction in TNF-α was observed between the two groups. The present findings therefore, in combination with those by Panda et al. (39) using MoDCs, suggest that there may be an age-associated defect in the cytokine secretion pathways. *n-*3 PUFA or MUFA consumption could impact on cytokine signaling pathways and thus inhibit the secretion of resistin, as a consequence of the observed reduction in consumption of SFAs by the MED-diet intervention group and the confirmed intake of olive oil by the urinary HTS concentrations. Our study is however limited by the poor control group and therefore these data need validating in future studies with more stringent control over dietary intake and more frequent contact with the study participants. Since the dietary intervention was not blinded to the participants it is possible that both groups improved their diets, as a reduction in resistin has been correlated with the consumption of more nutritious diets (67, 68).

### Impact of Findings

The significant reduction in resistin production after the MED-diet intervention is of biological relevance since elevated resistin is associated with insulin resistance, CVD risk and obesity (69). Therefore, if only 1 year of dietary change to increase consumption of more phenolic-rich foods can reduce resistin levels to those observed in the healthy, young controls, this could have beneficial implications as a potential target for reducing the burden of these age-associated conditions. Further investigation into the mechanism for this reduction in resistin secretion would be of interest. This finding could also be relevant for the future investigation of drug targets to treat these conditions, such as the current pharmaceutical use of the PPARγ agonist [thiazolidinedione (TZD)] to treat insulin resistance in patients with T2D, which is associated with numerous adverse side effects such as body weight gain, fluid retention, heart failure, bone fractures, and increased risk of bladder cancer (70). Patel et al. (71) show that expression of resistin by human macrophages can be reduced with extensive (96 h), but not short term (24 h), exposure to rosiglitazone, which is a TZD. Thus, if the public could be persuaded to adopt aspects of the MED diet there may be potential for the age-associated increase in resistin, as seen here, to be prevented, and thus the need for such drugs to improve insulin sensitivity might be reduced.

In summary, the finding of age-associated impairments in DCs, and that dietary intervention reduced the age-associated increases in resistin secretion represents both methodological improvements and novel findings in the field of immunonutrition and as such provides the basis for further investigation of the MED diet for immunomodulation, particularly in terms of increased adipokine secretion, with potential pharmaceutical implications.

### Recommendations for Future Work

To further validate our findings the next step includes testing the MED diet on antigen-specific immune responses in elderly subjects. This could be achieved by taking blood samples, including serum, pre- and one postvaccination (72, 73) with for example, the seasonal influenza vaccine, after MED-diet intervention for 1 year. Vaccine antigen serum antibody levels and antigen-specific memory T cell recall responses could be measured and compared pre- and post-dietary intervention. The overall design of the Nu-AGE study could be improved through, for example, the use of multiple additional biomarkers to assess dietary compliance, and the collection of more frequent blood samples would have been advantageous in providing more data points and in demonstrating reproducibility of the results. The inclusion of a much more stringent control diet group would also be necessary, as well as more frequent contact with the MED-diet intervention subjects, with the provision of more of the food components to the subjects within the study center, to both encourage and monitor changes in dietary intake.

## Ethics Statement

This study was carried out in accordance with the recommendations of the University of East Anglia Research Ethics Committee (UEA-REC) with written informed consent from all subjects. All subjects gave written informed consent in accordance with the Declaration of Helsinki. The Im-AGE study received ethical approval from the NRES South West – Cornwall and Plymouth Research Ethics Committee. The Nu-AGE study was approved by the NRES Committee East of England – Norfolk.

## Author Contributions

SJC, SRC, MM, CN and KI designed experiments. SJC and MM collected samples and data. SJC analyzed and carried out statistical analysis of data and with SRC interpreted the data. SJC and SRC prepared the manuscript; the content of this manuscript first appeared in SJC’s PhD thesis (74); the only medium within which these data have appeared. SRC and CN supervised the research.

## Conflict of Interest Statement

The authors declare that the research was conducted in the absence of any commercial or financial relationships that could be construed as a potential conflict of interest.
