# Supplementary material for: Age-Associated Decline in Dendritic Cell Function and the Impact of Mediterranean Diet Intervention in Elderly Subjects
Source: Front Nutr. 2017 Dec 19;4:65. doi: 10.3389/fnut.2017.00065 (PMC5742184; doi:10.3389/fnut.2017.00065)
Supplement: Supplementary file 1 [file Table_1.DOCX]

Supplementary Material

The impact of ageing and Mediterranean diet on dendritic cell populations and function in elderly individuals

Clements, S.J^1,2^, Maijo, M^1^, Ivory, K^1^., Nicoletti, C^1,3^. & Carding, S.R^1,2*^

*** Correspondence:** Professor Simon R Carding: (e) Simon.Carding@quadram.ac.uk

# Supplementary Figures and Tables

| **Food group** | **Quantity required in dietary intervention** |
| --- | --- |
| Whole grains | 6 servings per day; 1 serving=25 g bread, 50 g breakfast cereal |
| Fruits | 2 servings per day; 1 serving=1 apple, 1 banana, 8 small plums |
| Vegetables and legumes | 330 g per day, once per week 200 g legumes |
| Dairy and cheese | 500 ml dairy per day (of which 30 g cheese) |
| Fish and other seafood | 2 times per week; 1 portion=125 g |
| Meat and poultry | 4 times per week; 1 portion=125 g |
| Nuts | 2 times per week; 20 g portion |
| Potatoes, pasta and rice | 150 g per day; 80 g (raw weight) whole grain rice or pasta at least twice a week |
| Eggs | 2–4 times per week |
| Oil or fat | 20 g oil per day, 30 g margarine per day; maximum of 50 g fat per day. Should be olive oil and low fat margarine rich in MUFA and PUFA |
| Alcohol | Maximum of 1–2 glasses per day for men, and 1 glass per day for women. Preferably red wine, if not abstain |
| Fluid | 1.5 litre per day, including milk |
| Salt | Reduce added salt, and intake of ready meals (soups, gravy, sauce) |
| Sugar | Limit consumption of sugar and sweetened drinks (replace with fruit or yoghurt, no/reduce sugar in tea or coffee). |

Supplementary Table 1. Quantitative dietary guidelines given to study participants whom were allocated to the intervention arm of the study.

|  | | **Nu-Age cohort pre-intervention** | **Nu-AGE cohort post-intervention** |
| --- | --- | --- | --- |
| **Body weight (g)** | Mean (SD) | 74785 (13704) | 74200 (13490) |
|  | Range | 47731–128624 | 48007–119472 |
| **Fat mass (g)** | Mean (SD) | 23711 (8099) | 23119 (8212) |
|  | Range | 6656–50415 | 5559–55850 |
| **Lean mass (g)** | Mean (SD) | 48836 (10276) | 48847 (10125) |
|  | Range | 31910–81108 | 32543–78447 |
| **Bone mineral content (BMC) (g)** | Mean (SD) | 2238 (493.3) | 2243 (498.7) |
|  | Range | 1367–3841 | 1320–3903 |
| **Soft tissue (g)** | Mean (SD) | 72547 (13377) | 72007 (13173) |
|  | Range | 46113–125287 | 46365–116070 |
| **Fat mass (%)** | Mean (SD) | 32.5 (8.351) | 31.89 (8.564) |
|  | Range | 11.67–49.9 | 9.864–51.26 |
| **Regional fat mass (%)** | Mean (SD) | 31.55 (8.194) | 30.95 (8.405) |
|  | Range | 11.26–48.81 | 9.506–50.22 |
| **Bone mineral density (BMD) (g/cm^2^)** | Mean (SD) | 1.067 (0.1293) | 1.068 (0.1273) |
|  | Range | 0.816–1.512 | 0.8117–1.476 |
| **T score** | Mean (SD) | -0.8913 (1.245) | -0.9011 (1.202) |
|  | Range | -3.287–4.054 | -3.336–2.967 |
| **Fat mass/ lean mass** | Mean (SD) | 0.5039 (0.1827) | 0.4914 (0.1859) |
|  | Range | 0.1322–0.9959 | 0.1094–1.052 |

Supplementary Table 2 Dual x-ray bone densitometry (DXA) results for all Nu-AGE subjects at baseline (n=272), showing mean, standard deviation (SD) and range for each variable.

|  | **Control (n=57)** | **MED Diet (n=65)** |
| --- | --- | --- |
| **Pre-intervention** | | |
| Mean (SD) | 5.8 (1.6) | 5.5 (1.7) |
| Median | 6.0 | 6.0 |
| Range | 3–10 | 2–10 |
| **Post-intervention** | | |
| Mean (SD) | 4.7 (1.4) | 5.1 (1.7) |
| Median | 5.0 | 5.0 |
| Range | 1–9 | 2–7 |

**Supplementary Table 3. Mean and median values for Mediterranean diet scores for the control and MED diet groups, at pre- and post-intervention.**


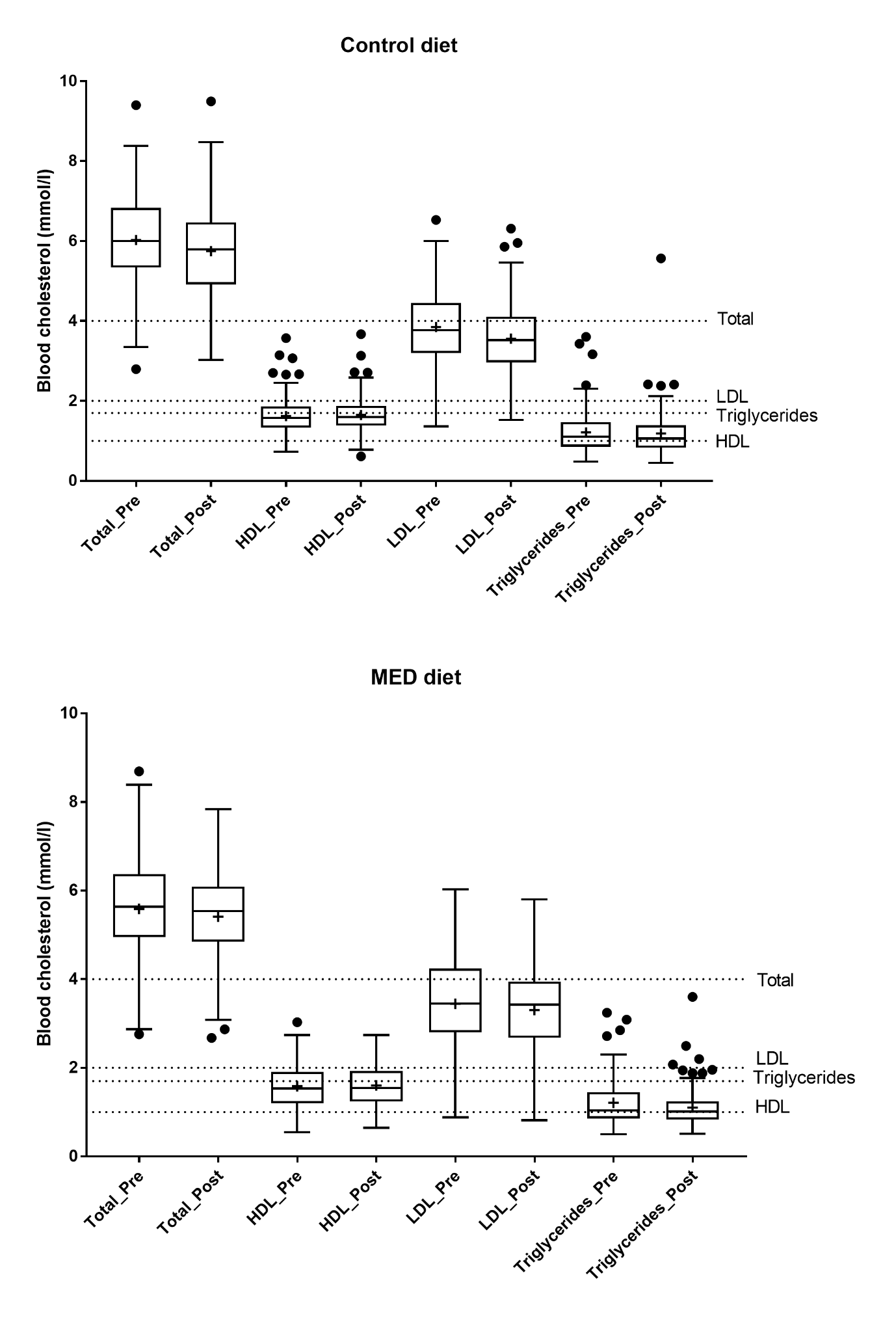


Supplementary Figure 1. Plasma lipid concentrations at pre- and post-intervention for all subjects, separated by allocated diet group. Plasma concentrations for all Nu-AGE subjects in the control (top graph) and MED-diet group (lower graph) are shown as box and whisker plots which extend from the 25^th^ to the 75^th^ percentiles, with the horizontal line representing the median and the plus (+) representing the mean. Whiskers are determined using Tukey’s method, which uses the 25^th^ and 75^th^ percentile plus 1.5 times the interquartile range (IQR). The dotted lines indicate the British Heart Foundation’s recommended levels of blood cholesterol and triglycerides; total cholesterol <4 mmol/l, LDL cholesterol <2 mmol/l, triglycerides <1.7 mmol/l and HDL cholesterol >1 mmol/l ([BHF, 2017](#_ENREF_49)). n=134, control group; n=138, MED-diet group.


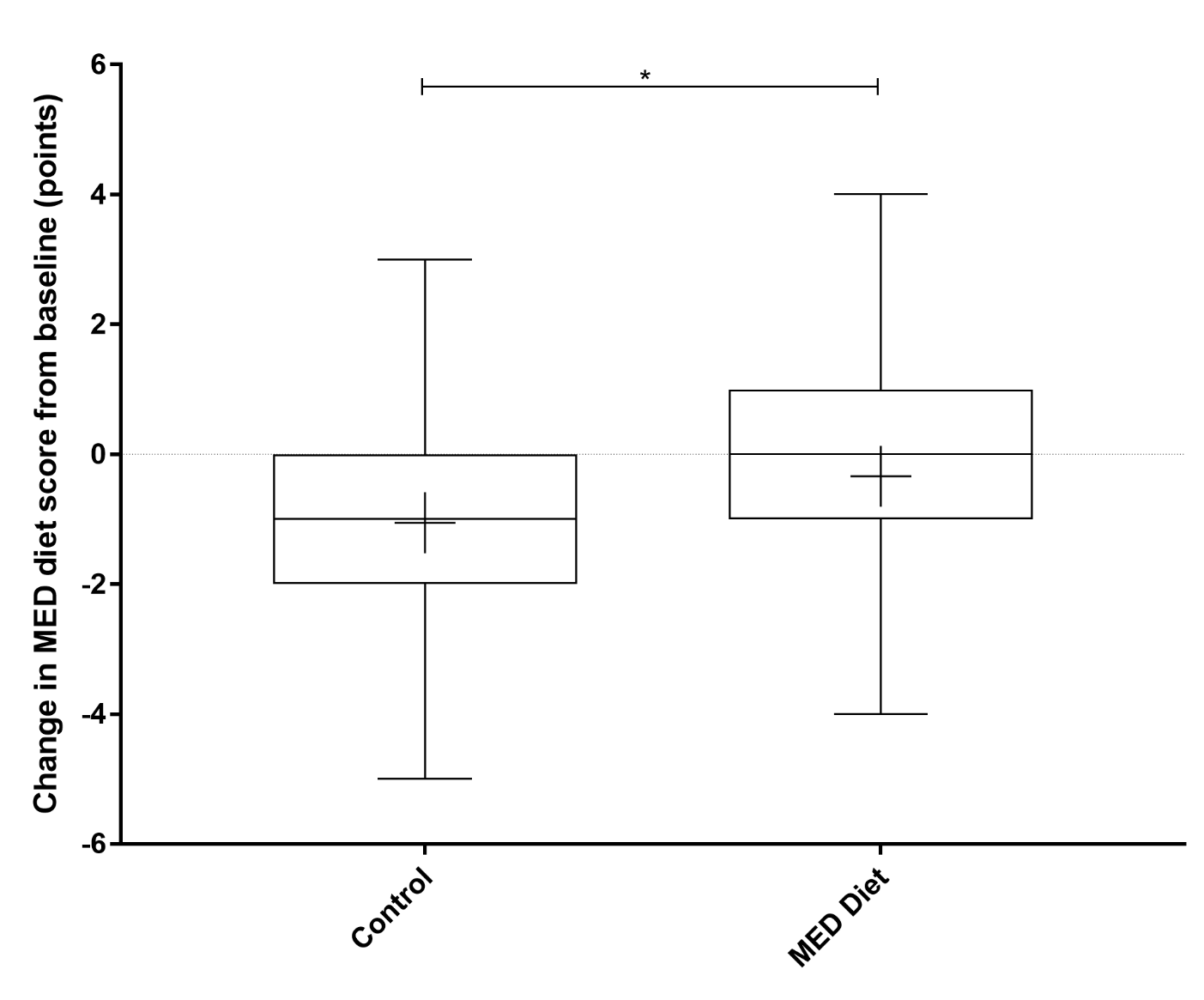


Supplementary Figure 2. Change in Mediterranean diet scores from baseline. Shown as box and whisker plots which extend to the 25^th^ and 75^th^ percentiles, with the mean change from baseline represented by the plus (+) and median change by the horizontal line through the box plots. Whiskers are determined using Tukey’s method, which uses the 25^th^ and 75^th^ percentile, plus 1.5 times the interquartile range (IQR). An unpaired t-test was used to determine whether the changes in the diet score differed between the two groups. * P <0.05.


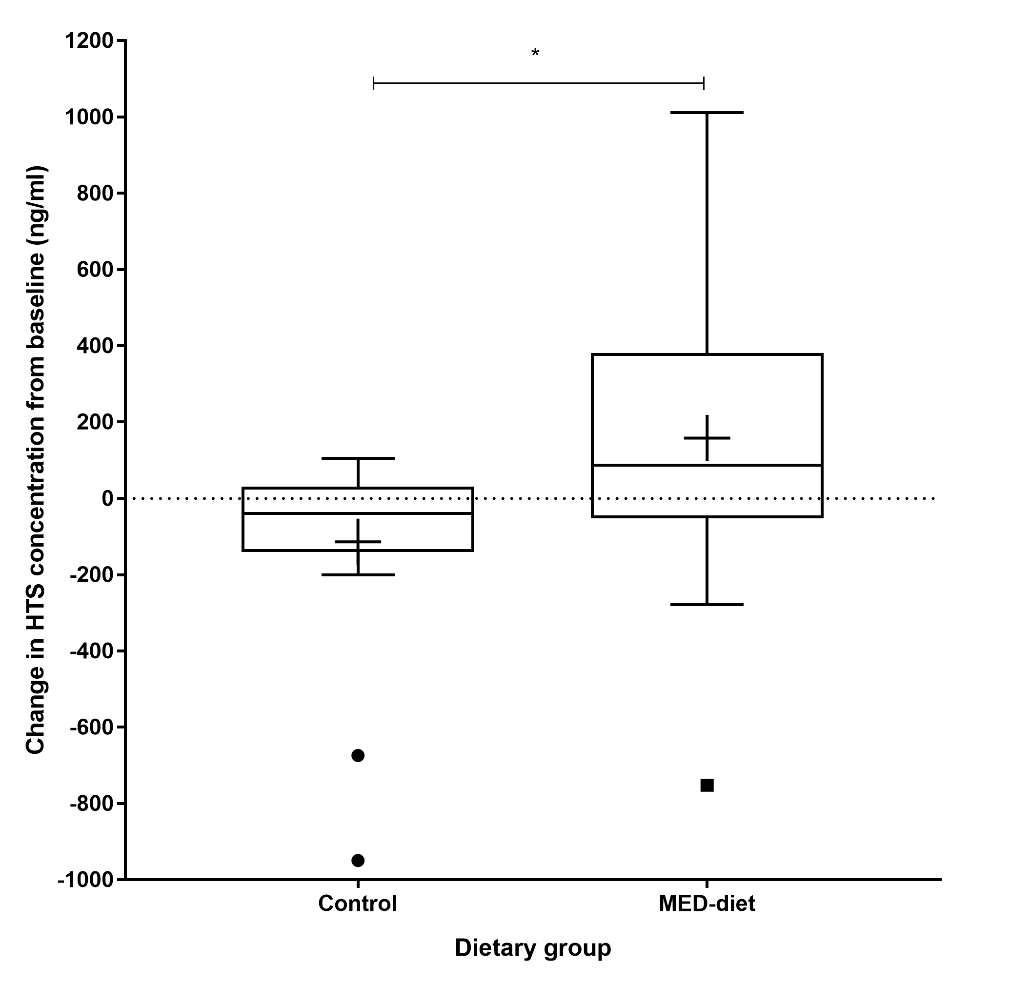


**Supplementary Figure 3. Change in hydroxytyrosol sulphate (HTS) (ng/ml) from baseline, determined by** **High Performance Liquid Chromatography-Mass Spectrometry (HPLC-MS/MS) analysis of urine samples.** The column used was 2.7µm Supelco Ascentis Express C18 (150 mm x 4.6 mm i.d) with a mobile phase of water/ 0.1% formic acid as solvent A, and acetonitrile/ 0.1% formic acid as solvent B, at a temperature of 40°C. The HPLC gradient began with 5% of solvent B, followed by an increase to 55% at 11 minutes for one minute, then increased to 95% for a further four minutes, and was then returned to the initial conditions in 0.1 minutes, with a re-equilibration time of 22 minutes. The flow rate was 600 µl/ minute and the injection volume was 10 µl. The standard used was Hydroxy Tyrosol 3- sulphate sodium salt (Toronto Research Chemical Inc) which has a molecular weight of 232 g. Hydroxytyrosol was detected and quantified according to its ion fragmentation in the tandem MS/MS using multiple reaction monitoring (MRM) mode, and ionisation was performed in the negative mode. Taxifolin was used as the internal standard, the product ion had a molecular weight of 125 m/z. Shown as box and whisker plots which extend to the 25^th^ and 75^th^ percentiles, with the mean change from baseline represented by the plus (+) and median change by the horizontal line through the box plots. Whiskers are determined using Tukey’s method, which uses the 25^th^ and 75^th^ percentile, plus 1.5 times the interquartile range (IQR). An unpaired t-test was used to determine whether the changes in the HTS concentration differed between the two groups.***** P <0.05.

**
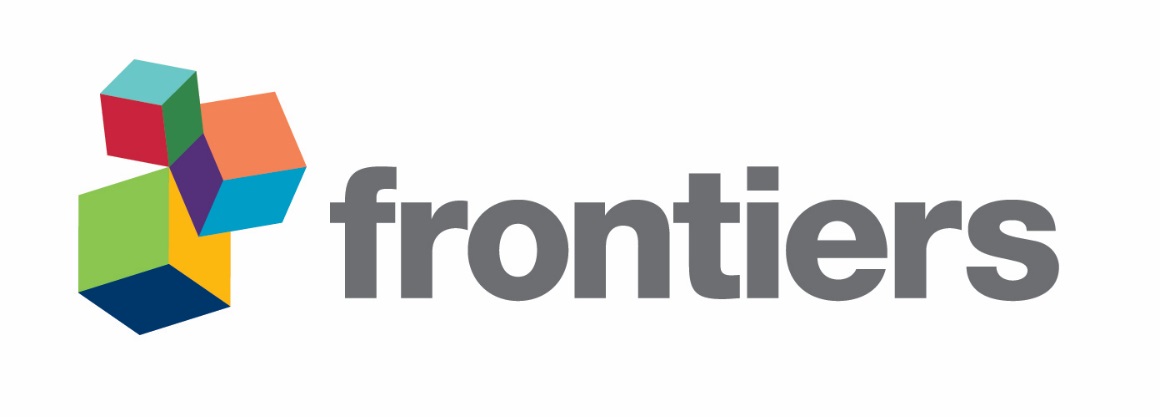
**
